# Effectiveness of the 5As Model-Based Transitional Care Program among Chinese Patients with Type B Aortic Dissection Post-TEVAR: A Randomized Controlled Trial

**DOI:** 10.31083/j.rcm2509347

**Published:** 2024-09-24

**Authors:** Jianxin Tu, Jing Zhou, Xiumao Li, Qin Zhang, Mingxian Luo, Jiamei Zhou

**Affiliations:** ^1^Abdominal Oncology Department, The Second Affiliated Hospital of Zunyi Medical University, 563000 Zunyi, Guizhou, China; ^2^Nursing School, Zunyi Medical University, 563002 Zunyi, Guizhou, China; ^3^Cardiovascular Surgery Department, Affiliated Hospital of Zunyi Medical University, 563000 Zunyi, Guizhou, China; ^4^Nursing Department, Affiliated Hospital of Zunyi Medical University, 563000 Zunyi, Guizhou, China

**Keywords:** Stanford type B aortic dissection, disease management, transitional care, 5As model, thoracic endovascular aortic repair

## Abstract

**Background:**

Thoracic aortic endovascular repair (TEVAR) is the primary treatment for Stanford type B aortic dissection (type B AD). However, patients often encounter significant difficulties post-TEVAR that endanger their safety when transitioning from hospital- to home-based care. Moreover, information on the ideal transitional care for patients with type B AD post-TEVAR is scarce in China. This single-masked randomized clinical trial aimed to assess the effectiveness of the Assess, Advise, Agree, Assist, and Arrange (5As) model-based transitional care in improving discharge preparation level and transitional care quality post-TEVAR among patients with type B AD in China.

**Methods:**

This study was conducted at a hospital in China between January 2021 and October 2021. Patients with type B AD were randomly divided into intervention and control groups. Participants in the intervention group received the 5As model-based transitional nursing care. The 5As model is an evidence-based intervention strategy comprising: (1) Assess: assessing the preoperative cardiovascular risk behavior of patients with AD. (2) Advise: making suggestions according to the risk behaviors of the patients. (3) Agree: reaching a consensus on goals and action plans by making decisions with the patients and their families. (4) Assist: assisting patients in solving obstacles to implementing health plans. (5) Arrange: arranging follow-up visits according to the actual situation of the patients and guiding them in adhering to a schedule. The control group received the usual nursing care for the same duration and number of follow-up visits. A trained research nurse collected all the baseline data of the patients on admission, assessed discharge readiness level (using the Readiness for Hospital Discharge Scale) on the day of discharge, and collected transitional quality of care (by the Care Transition Measure-15) data on day 30 after discharge.

**Results:**

Overall, 72 patients with type B AD were recruited. Discharge readiness level and transitional care quality in the intervention group were significantly superior to those in the control group.

**Conclusions:**

This study showed that the 5As model-based transitional care program can effectively promote discharge readiness and transitional care quality of patients with type B AD post-TEVAR.

**Clinical Trial Registration:**

The Chinese Clinical Trial Registry Center: ChiCTR2200060797 (https://www.chictr.org.cn/showproj.html?proj=167403).

## 1. Introduction

Thoracic aortic endovascular repair (TEVAR) is the primary treatment for Stanford type B aortic dissection (type B AD). Most patients with type B AD prefer TEVAR to conservative pharmacological therapy [1]. Data from the China National Center for Cardiovascular Disease showed that 75% of the patients with type B AD underwent TEVAR in 2019 [2]. However, community medical staff lack the knowledge of aortic dissection management and related professional knowledge and skills required during patients’ discharge and transitioning from hospital to home post-TEVAR. Ultimately, this can result in the inability of the patients to manage the disease at home following discharge [3, 4, 5], leading to an increased probability of early hospital readmission (EHR) and compromised patient safety. A study has shown that the EHR rate among patients with type B AD reached 25.1%, with the main reasons noted as endoleaks and continuous dilation of the aneurysm sac post-TEVAR, requiring endovascular and/or open thoracic aortic repair [6, 7]. One of the main reasons for recurrence is that patients lack good health-promoting behaviors with poorly controlled blood pressure [8].

In addition, a previous survey on quality of life and health behaviors in patients with aortic dissection [9] and similar study showed that the quality of life of the survivors of type B AD is suboptimal [10]. However, studies on the transitional care of patients with type B AD are limited, while studies on the traditional models of continuing care have either reported limited interventions or focused on only one aspect of care [11, 12]. Patients are passively involved in transitional care with low levels of active participation and a lack of objective indicators and feedback mechanisms for evaluating the effects of transitional care [13]. Furthermore, interventions typically begin at or after discharge, and the individual and ideal transitional care outcome indices, including preparation for discharge, quality of patient-centered transitional care, and utilization of health care services, have yet to be thoroughly examined. Hansen *et al*. [14] suggested predischarge interventions, including patient education, discharge plans, and drug regulations, of which predischarge patient education has become an important part of transitional care. Furthermore, the advancement of network technology and the increasing popularity of mobile phones have made transitional care more effective and accessible.

This study aimed to improve the readiness of patients for hospital discharge and the quality of transitional care using the Assess, Advise, Agree, Assist, and Arrange (5As) model, an evidence-based intervention strategy endorsed by the Centers for Medicare and Medicaid Services and the United States Preventive Services Task Force that can serve as a theoretical framework for assessing patients’ health behaviors, readiness to change, and the implementation and evaluation of behavioral and lifestyle interventions [15], thus offering a time-saving and efficient way to counsel patients and reduce the likelihood of type B AD-related readmissions.

## 2. Materials and Methods

### 2.1 Design and Sample

Research participants were recruited from the Affiliated Hospital of Zunyi Medical University between January 2021 and October 2021. A researcher contacted eligible patients and those who met the inclusion criteria at the time of admission and provided written informed consent were enrolled. The inclusion criteria were (1) age ≥18 years; (2) diagnosis based on the diagnostic criteria of AD in “Braunwald Cardiology” [16], in which a diagnosis of type B AD was based on clinical manifestations, cardiac ultrasound, and computed tomography angiography; (3) ability to use WeChat; (4) provision of written informed consent. The exclusion criteria were (1) the presence of cognitive or psychiatric problems, (2) transfer to another medical facility, and (3) inability to use emails. All protocols were performed in accordance with relevant guidelines and regulations.

The sample size was calculated using the formula shown in Eqn. 1 [17], as previously described [18].

(1) $$n\,1=n\,2=2\,{\left[\left(u_{\alpha }+u_{\beta }\right)/\left(\delta /\sigma \right)\right]}^{2}+0.25\,u\,\alpha^{2}$$

where $u_{\alpha }=1.96;u_{\beta }=1.282;\delta /\sigma =0.85;$ and n⁢1=n⁢2=30.

The calculated sample size was 60. Allowing for a 20% dropout rate, each group comprised 36 individuals. The patients were randomly divided into intervention and control groups using the random number table method. The specific randomized scheme was as follows: (1) numbering: 72 patients were numbered according to the order of admission; (2) generating random numbers: 72 random numbers (repeated numbers skipped) were successively selected from the second row and first column of the random number table as patient numbers, and then the random numbers were ranked successively in ascending order; (3) random grouping: the rank of the random numbers was 1–36 for the intervention group and 37–72 for the control group.

### 2.2 Study Protocol and Intervention

The intervention group received the 5As model-based transitional nursing care, created by the research group through expert meetings based on literature analysis and the development of relevant standards. The program’s draft was reviewed by nine experts (four cardiovascular specialists, four cardiovascular nursing specialists, and one chronic illness management specialist). Experts were invited if they met the following criteria: (1) at least 10 years of relevant work experience and (2) possessed a doctorate degree or held a prominent professional position. Based on their suggestions, the program was modified and improved. Table 1 presents the final transitional care program.

**Table 1.** **Transitional care program**.

5As	Goal	Implementer	Intervention
Assess (20–30 min)	Documentation of the initial status of patients’ health behaviors.	Registered nurse and doctor.	(1) Ask patients to complete a lifestyle and health assessment questionnaire; review weight, BMI, BP, medical history, and AD risk factors (obesity, drinking, sedentary lifestyle, unhealthy diet, and smoking).
			(2) “Health Promotion Behavior Education Handbook for AD” part 1: Overview of AD, classification, clinical signs and symptoms, treatment, disease risk factors, admission preparation, care goals, and patient and family roles and responsibilities in health management.
			Methods: Patients were provided a guidebook and instructed to read it beforehand. Playing videos on the iPad. One-on-one and face-to-face instructions were available. Response and discussion: WeChat was used to share learning materials and answer inquiries.
Advise (30–40 min)	Provider-based counseling, including specific lifestyle behavior suggestions, should be documented in the medical records of patients with AD. Doctors, nurses, dieticians, behavioral counselors, physical therapists, patients, and family members jointly develop personalized discharge plans.	Registered nurse, doctor, dietician, behavioral counselor, and physical therapist.	(1) Assess the patient’s discharge readiness within 2 days before discharge. Based on the detailed understanding of the patient’s condition throughout the diagnosis and treatment process, combined with the information collected by the evaluation, the researcher informed the patient and caregivers of the patient’s risk factors, such as diabetes, hypertension, smoking, lack of exercise, overweight, unhealthy diet, and alcoholism.
			(2) “Health Promotion Behavior Education Handbook” for AD Part 2 and Part 3: healthy lifestyle for patients with AD, daily life guidance, methods of measuring and recording blood pressure.
			Methods: Using the iPad to play videos. One-on-one and face-to-face instructions were available. Response and discussion: WeChat was used to share learning materials and answer inquiries.
			(3) Depending on the participants’ physical condition and functional status, a stepwise graded exercise approach was followed, including improving functional activities and increasing muscle strength.
			Provide clear, specific, personalized advice on behavioral/lifestyle changes without judgment. Provide and review lifestyle recommendations at each visit: quit smoking, engage in physical activity, such as walking for 30 minutes, 4–7 days per week; weight loss with a body mass index >28 kg/m^2^; dietary sodium limit <1500 mg/day. A Mediterranean diet is recommended.
Agree (20–30 min)	Record the patient’s goals (short-term and long-term) and track the progress by reviewing and discussing with the patient at each visit.	Registered nurse, dietician, behavioral counselor, and physical therapist.	Work with patients to set realistic diet and behavioral change-related goals. Ask open-ended questions to assess the patient’s motivation and confidence to change. Work with patients to identify and define accurate and achievable short-term goals. Example: 1 month: reduce daily saturated fat amount; increase the daily portion of fruits/vegetables. The two sides must agree on long-term goals.
Assist (25–35 min)	Evaluation of patient obedience and compliance with the diet and lifestyle/behavioral changes demonstrated in the AD health promotion behavior questionnaire.	Registered nurse and pharmacist.	Assist patients in identifying and overcoming obstacles to the diet plan’s implementation. Help patients adjust medication dosage according to blood pressure and other lifestyle changes. Problem-solving techniques and family support should be used. At each patient visit, continue to offer advice/counseling support.
Arrange	Follow-up patient assessment on the 30th and 90th days post-discharge. Improve the rate of patients returning to the hospital for review.	Registered nurse.	After the patient is discharged from the hospital, WeChat is used to send learning materials and answer inquiries once a week, four times a month for 3 months.
			Arrange follow-up visits to provide assistance. According to the actual situation of the patients, choose the appropriate follow-up methods, such as via telephone or email. Schedule hospital reviews for patient support and assess patients’ compliance with the plan.

AD, aortic dissection; BMI, body mass index; BP, blood pressure.

In the control group, the participants received normal care in accordance with hospital guidelines. Routine nursing care after admission was performed, as well as general evaluation, general hospital propaganda, such as the environment department personnel arrangements within the department, family visitation rules during the coronavirus disease 2019 outbreak, and education. During hospitalization, patients and caregivers were informed about the purpose of the inspection and time according to the division within the conventional nursing program. The patients were introduced to AD-related knowledge, purpose, function, and key points regarding various treatments. When discharged, the patients and caregivers were guided to undergo the discharge procedures. They were also informed about relevant rehabilitation knowledge, including the type of medicine, treatment record, post-discharge medications, diet, and exercise suggestions. Finally, the importance of timely follow-up visits was emphasized.

### 2.3 Quality Control Method 

Several strategies were adopted to ensure intervention fidelity. First, participants were instructed to strictly adhere to the project’s research procedures and training provided by planned implementers in the department meeting room through lectures that impart scientific knowledge and skills. The scientific expertise includes diet, exercise, and blood pressure management; skill learning involves the 5As consulting method, which helps the medical staff to complete the five consulting steps in a short period: (1) assessment of the preoperative cardiovascular risk behavior of the patients with AD; (2) making suggestions according to the risk behaviors of the patients; (3) reaching an agreement concerning the goals and action plans by making joint decisions with the patients and their families; (4) assisting patients in solving their obstacles in the implementation of health plans; (5) arranging follow-up visits according to the actual situation of the patients, guiding them to follow-up within the specified time. Second, we standardized the management of patient education and telephone follow-up projects, established a self-management health manual for patients with AD, and established systematic telephone follow-up records. Third, a checklist for the intervention was created, and each item was checked upon completion. Fourth, the study group met once a week to discuss the status of the study and assess whether the interventions were administered as planned.

### 2.4 Outcome Variables and Measures 

Discharge readiness level and transitional care quality were assessed using the appropriate instruments and tools. We conducted a preliminary experiment with 20 patients with AD before the main study to test the feasibility of the study methodology and establish the reliability of the scale.

#### 2.4.1 Readiness for Hospital Discharge 

The Readiness for Hospital Discharge Scale (RHDS) was developed by Weiss *et al*. [19] to assess hospital discharge readiness levels. The Chinese version of the scale was translated and revised by Lin *et al*. [20]. This study adopted a questionnaire survey method with 12 items and three dimensions: Personal status, coping ability, and expectation support. Each item can be scored from 0 to 10. The total score (range: 0–120) was calculated by adding the scores of the three subscales. Higher scores indicate a higher degree of discharge preparation. The content validity index of the Chinese RHDS is 0.88, the total Cronbach’s α is 0.89, and the subscale Cronbach’s α range is 0.80–1.00. In this study, Cronbach’s α was 0.791, and the test–retest reliability was 0.820.

#### 2.4.2 Transitional Care Quality 

The Care Transition Measure-15 (CTM-15) [21] was used to measure the quality of transitional care. It consists of 15 items and four subscales. Each item is scored on a scale of 1 to 4, with 1 indicating “highly disagree”, 4 “highly agree”, and 0, “do not know/do not remember/do not apply”. Higher scores indicate a better quality of transitional care. The scale has good construct and discriminant validity, with a Cronbach’s alpha of 0.93. This study used the Chinese version of the CTM-15 to evaluate the quality of transitional nursing care [22]. The Cronbach’s α was 0.830, and test-retest reliability was 0.802 in the present study.

### 2.5 Data Collection 

A trained nurse collected baseline data from the patients upon admission, evaluated the discharge readiness scale on the day of discharge, and collected the transitional care quality scale scores on the 30th day after discharge.

### 2.6 Statistical Analysis 

Data were analyzed using SPSS WIN (version 18.0, IBM Corp., Armonk, NY, USA). General categorical data, such as sex, marital status, medical insurance type, education level, and occupation, are presented as frequencies and percentages and were analyzed using the chi-square test or Fisher’s exact probability method. Age, length of hospital stay, hospitalization expenses, and other metric data are presented as the mean, standard deviation, or quartile and were analyzed using the independent samples *t*-test or rank-sum test. The discharge readiness and transitional care quality scores of the two groups are described as the median and interquartile interval or the mean and standard deviation. The differences in the transitional care quality scores between the two groups were analyzed using a two-sample independent *t*-test or rank-sum test. The selected inspection level of the statistical results was α = 0.05, and a *p*-value < 0.05 indicated statistical significance.

## 3. Results

### 3.1 Study Population

**Fig. 1.**
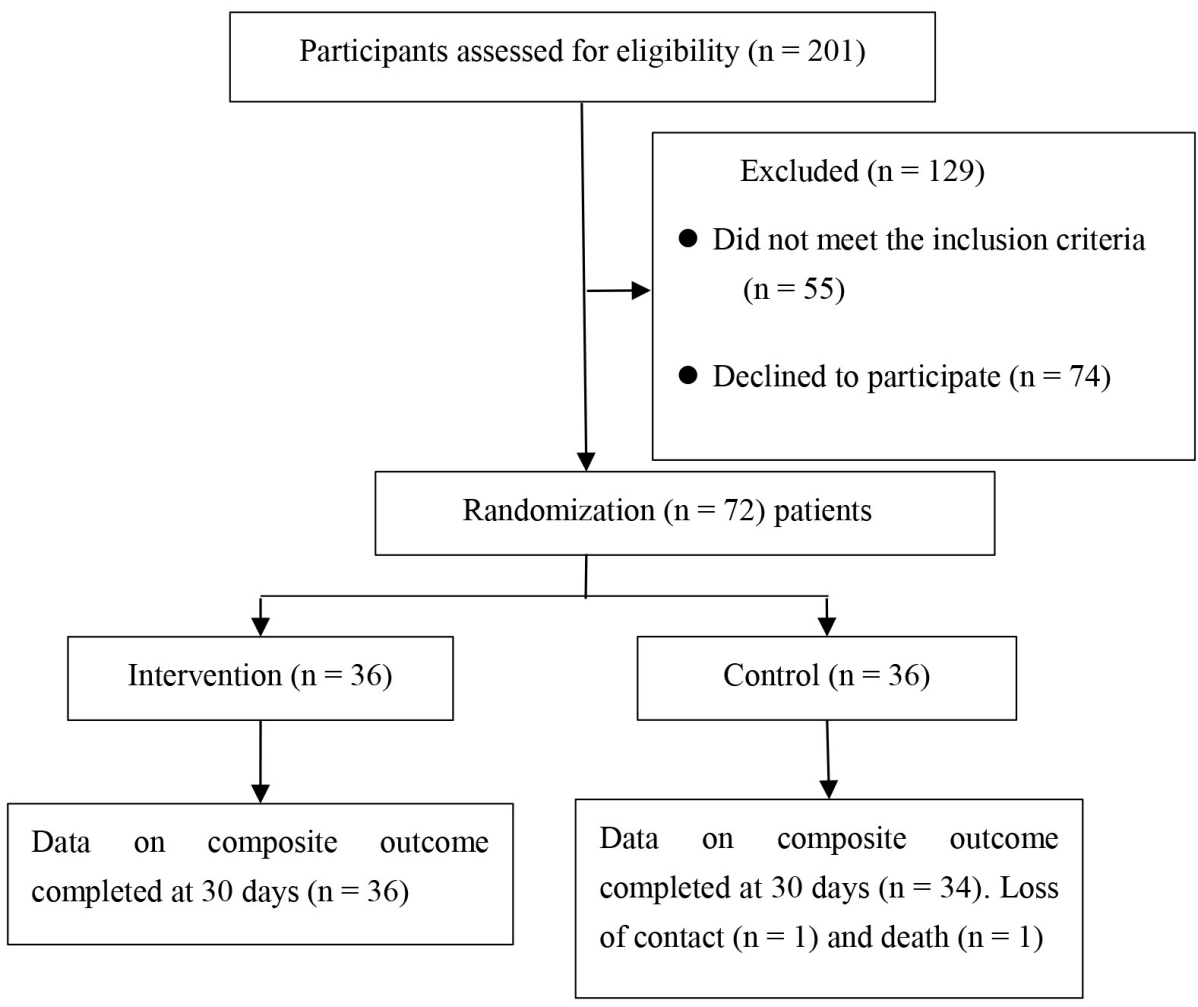
**Study flowchart**.

In the 201 pairs of screened participants, 55 patients were ineligible, and 74 patients declined to participate. A total of 72 pairs of eligible participants agreed to participate in this study and were randomly assigned to the intervention group (n = 36) or the control group (n = 36). During the research period, two patients in the control group were lost on the 30th day after discharge, with a follow-up rate of 5.56%. In the intervention and the control groups, 36 and 34 patients completed this research, respectively. The comprehensive standard flowchart of this research is shown in Fig. 1.

### 3.2 Participants’ Demographic Characteristics 

Table 2 shows the characteristics of the participants in the two groups. There were no remarkable differences in the characteristics of the participants in the intervention and control groups, except for the length of hospital stay (*p* > 0.05).

**Table 2.** **Characteristics of the participants**.

		Intervention (n = 36) ($\bar{x}$ ± s)/N (%)	Control (n = 34) ($\bar{x}$ ± s)/N (%)	χ ^2^ */Z/t*	*p*-value
Age		53.89 ± 11.39	55.41 ± 2.16	0.532^1^	0.597
Sex	Male	30 (83.3)	24 (29.4)	2.319^2^	0.128
	Female	6 (16.7)	10 (70.6)		
Marital status	Married	32 (88.9)	32 (94.1)	-^3^	0.674
	Divorced/widowed	4 (11.1)	2 (38.2)		
Educational level	Primary school and below	15 (41.7)	10 (8.8)	3.065^4^	0.383
	Junior high school	14 (38.9)	14 (41.2)		
	Senior high school	2 (5.6)	6 (38.2)		
	College or university and above	5 (13.9)	4 (11.8)		
Work status	Farmer	13 (36.1)	14 (41.2)	2.840^4^	0.585
	Employed	7 (19.4)	5 (14.7)		
	Retired	1 (2.8)	4 (11.8)		
	Unemployed	13 (36.1)	10 (29.4)		
	Merchant	2 (5.6)	1 (2.9)		
Healthcare payment type	Medical insurance for urban residents	6 (16.7)	4 (11.8)	1.653^4^	0.799
	Medical insurance for employees	6 (16.7)	8 (23.5)		
	New rural cooperative medical scheme	22 (61.1)	21 (61.8)		
	Commercial insurance	1 (2.8)	1 (2.9)		
	Self-paying	1 (2.8)	0 (0)		
Per capita monthly income (¥, Yuan, 1 RMB = 0.141 USD)	<1000	9 (25)	9 (26.5)	2.443^4^	0.486
	1000–3000	11 (30.6)	13 (38.2)		
	3001–5000	8 (22.2)	9 (26.5)		
	>5000	8 (22.2)	3 (8.8)		
Living status	Living alone	4 (11.1)	2 (5.9)	0.690^4^	0.708
	Living with partner	23 (63.9)	24 (70.6)		
	Living with children	9 (25)	8 (23.5)		
Hypertension	Yes	30 (83.3)	30 (88.2)	-^3^	0.736
	No	6 (16.7)	4 (11.8)		
Hospital stay (days)		9.81 (8, 11)	13.38 (9.75, 17.25)	–2.889^2^	0.004
Hospitalization costs (¥, ten thousand Yuan, 1 RMB = 0.141 USD)		7.95 ± 2.69	9.18 ± 3.15	1.751^1^	0.084

^1^Independent samples *t*-test; ^2^Wilcoxon–Mann–Whitney U test; ^3^Fisher’s exact test; ^4^Pearson’s chi-square test.

### 3.3 Effect of the 5As Model-Based Transitional Care Program on Discharge Readiness 

The results presented in Table 3 show that the intervention group had remarkably higher values than the control group in terms of personal status, coping ability, expected support, and total RHDS scores (*p* < 0.05).

**Table 3.** **Contrast of the discharge readiness between the patients in the intervention and control groups**.

	Intervention group (n = 36) mean ± SD	Control group (n = 34) mean ± SD	*t*	*p*-value
Personal status	16.75 ± 5.07	14.18 ± 2.97	2.571	0.012
Coping ability	37.97 ± 4.24	32.79 ± 6.20	4.098	<0.001
Expected support	31.17 ± 4.3	26.65 ± 7.06	3.255	0.02
Total	85.89 ± 10.93	73.62 ± 10.94	4.692	<0.001

### 3.4 Effect of the 5As Model-Based Transitional Care Program on Transitional Care Quality 

Table 4 shows the scores for critical understanding, importance of preference, management preparation, existence of a written and understandable care plan, and total score of transitional nursing care quality, all of which were significantly higher in the intervention group than in the control group (*p* < 0.05).

**Table 4.** **Comparison of the transitional care quality between the patients in the intervention and control groups**.

	Intervention group (n = 36) mean ± SD	Control group (n = 34) mean ± SD	*t*	*p*-value
Importance of preferences	8.44 ± 2.20	4.97 ± 2.75	5.86	<0.001
Management preparation	10.69 ± 2.49	6.06 ± 3.26	6.71	<0.001
Critical understanding	14.72 ± 3.49	7.59 ± 4.09	7.86	<0.001
Written and understandable care plan	6.61 ± 1.02	5.24 ± 1.46	4.59	<0.001
Total	40.47 ± 6.01	23.85 ± 6.66	10.97	<0.001

## 4. Discussion

Patients with type B AD face several disease management problems post-TEVAR, particularly when transitioning from hospital to home; thus, they were selected as the target population for our study. Our findings demonstrated the effectiveness of the transitional care program in reducing the hospital stay duration, enhancing discharge readiness, and enhancing transitional care quality in patients with type B AD.

### 4.1 Effectiveness of the 5As Model-Based Transitional Care Program on Hospital Discharge Readiness 

TEVAR has gained popularity owing to the advancement of medical technology, with a decrease in the average length of hospital stay, a reduction in the time to prepare for discharge, and prevention of inadequate preparation for discharge. Improving discharge preparation is one of the most effective measures against EHR [23]. Our findings showed that the 5As model-based transitional care program improved the discharge readiness of patients with type B AD, consistent with Xu *et al*. [24] and Peyrovi *et al*. [25]. The effectiveness of our findings can be attributed to several factors. First, based on the new definition of admission as preparation for discharge, early and timely assessment of AD risk factors and rapid discharge preparation-related interventions following admission provide patients with more time to prepare for hospital discharge and are associated with better outcomes [26]. Second, individualized and targeted health behavior recommendations were provided to the patients during hospitalization, and post-discharge health behavior goals were set according to the patient’s priorities. Ha Dinh *et al*. [27] showed that targeted interventions can encourage participants to receive more valuable information. Throughout the intervention, caregivers were encouraged to work with the patients to set discharge rehabilitation goals and strengthen their social support. Another important finding of our study was that the length of hospital stay was significantly shorter in the intervention group than in the control group. This may be related to our transition plan, including preoperative and early postoperative rehabilitation recommendations.

### 4.2 Effectiveness of the 5As Model-Based Transitional Care Program Regarding Transitional Care Quality 

The patient’s perspective is key to determining the quality of referral management. The Medical Research Institute emphasizes the need to evaluate the quality of transitional care from the patient’s perspective [28]. Therefore, our study used the CTM-15 to assess patients’ perceptions of the quality of transitional care. Our results suggest that scientifically designed transitional care plans can improve the quality of transitional care for patients with type B AD, consistent with earlier research findings [29, 30]. The success of this transitional care plan lies in filling the gap in the transition from hospital to home. The 5As model is an evidence-based intervention strategy that includes health education, coaching, counseling, and psychosocial support. Although there is no consensus on the composition of transitional care post-TEVAR, the participation of patients and medical staff is important. Intervention measures may include discharge planning, patient and family education, follow-up care, rehabilitation, and information exchange between nursing providers [31, 32]. Moreover, study has shown that trust-based relationships between patients and nurses can be established and maintained using appropriate methods with advanced nursing knowledge and skills [33]. Similarly, those conducting the interventions in this study received training through lectures on scientific knowledge and skills. Accordingly, patients could actively and accurately seek external help when faced with environmental changes and adverse stimuli to achieve the best adaptation state.

In addition, in our study, on the 90th day after discharge, the proportion of patients in the intervention group with controlled blood pressure at the recommended level (100–120/60–80 mmHg) was 75%, which was higher than in the control group (58.9%). These findings are similar to those reported by Carroll *et al*. [34]. Our previous study showed that although most patients administered their medication regularly, nearly 67.2% did not achieve a controlled blood pressure at the recommended level. Most patients were unaware of other blood pressure control methods besides the use of antihypertensive drugs, and many did not master the correct technique and timing of blood pressure measurement, leading to a deviation in blood pressure records; this may be one of the reasons for hospital readmissions [9]. In our study, patients were also trained in non-drug blood pressure control methods and blood pressure measurement skills. We invited pharmacists to participate, and the 5As were used to create a patient-centered model for counseling patients regarding blood pressure to increase the frequency of visits for those with poorly controlled hypertension.

### 4.3 Strengths and Limitations

One of the strengths of our study is that we considered poor blood pressure control in patients with type B AD. After discharge, pharmacists helped patients adjust their medication to control their blood pressure levels. Another advantage is interdisciplinary cooperation, which helps identify risk factors associated with a patient’s previous lifestyle early.

However, our study has a limitation. We could not measure the impact of the transitional care program on readmission and mortality rates. A larger sample size and longer follow-up period are needed to evaluate the effects of transitional care plans on readmission rates and mortality.

## 5. Conclusions

The 5As model-based transitional care program effectively promoted discharge readiness and the quality of transitional care in patients with type B AD post-TEVAR. These findings highlight the critical role of nurses in enhancing patient outcomes and the quality of care by providing a continuum of care from hospital to home. Future studies regarding the transitional care program should be performed with a larger sample size to achieve adequate statistical power to detect the effect of the intervention.

## Availability of Data and Materials

The datasets generated and analysed during the study are not publicly available as per the ethical approval for the study, but are available from the corresponding author on reasonable request.

## Abbreviations

TEVAR, thoracic aortic endovascular repair; EHR, early hospital readmission; RHDS, readiness for hospital discharge scale; type B AD, type B aortic dissection; CTM-15, care transition measure-15.
